# Comprehensive database of Chorismate synthase enzyme from shikimate pathway in pathogenic bacteria

**DOI:** 10.1186/2050-6511-14-29

**Published:** 2013-05-22

**Authors:** Prabakaran Pitchandi, Waheeta Hopper, Rathankar Rao

**Affiliations:** 1 Department of Bioinformatics, School of Bioengineering, Faculty of Engineering and Technology, SRM University, Kattankulathur, 603203, Tamil, Nadu, India; 2 Sr. Application Scientist, Apsara Innovations, #218/1, 2nd floor, Kammanahalli main road, 3rd block, Kalyana nagar, Bangalore 560 043, India

**Keywords:** Biological database, Shikimate pathway, Chorismate synthase, Pathogenic bacteria, Drug design, IC50

## Abstract

**Background:**

Infectious diseases are major public health problem. It is increasingly affecting more than 50 million people worldwide. Targeting shikimate pathway could be efficiently used for the development of broad spectrum antimicrobial compound against variety of infectious diseases. Chorismate synthase is an enzyme in shikimate pathway that catalyzes Phosphoenol pyruvate to chorismate in most of the prokaryotic bacteria. This step is crucial for its growth, since Chorismate acts as a precursor molecule for the synthesis of aromatic amino acids. Hence, we present a comprehensive database of Chorismate Synthase Database (CSDB) which is a manually curated database. It provides information on the sequence, structure and biological activity of chorismate synthase from shikimate pathway of pathogenic bacteria. Design of suitable inhibitors for this enzyme, hence could be a probable solution to destroy its proteomic machinery and thereby inhibit the bacterial growth.

**Description:**

The aim of this study was to characterise chorismate synthase enzyme belonging to pathogenic bacteria to analysis the functional and structural characterization of chorismate synthase is very important for both structure-based and ligand based drug design.

**Conclusions:**

The broad range of data easy to use user interface makes csdb.in a useful database for researchers in designing drugs.

## Background

Biomolecules databases, in general contain gene function, structure and localization of cell and chromosome. This also includes clinical effects of mutations, sequence and structural properties of proteins, domains, motifs and their functional roles in a protein and pathway information [1]. Targeting the seven enzymes of shikimate pathway could be an effective target for the development of antimicrobial and herbicidal compounds as it is a crucial pathway for synthesis of aromatic amino acid in bacteria and plants but not in mammals [2-7]. Chorismate synthase (CS) catalyzes conversion of 5- enolpyruvylshikimate 3-phosphate (EPSP) to chorismate, is the final step of shikimate pathway [3,8]. It is also an essential precursor for the synthesis of *p*-aminobenzoic acid and folate [9].

Chorismate synthase also plays a remarkable role in the biosynthesis of nucleotides. The reaction of chorismate synthase is unique in nature, involves a 1, 4 elimination of phosphate and loss of proton of the C-6 hydrogen. The formation of two out of three necessary double bonds to build an aromatic amino acid is aided by CS and activity of this enzyme requires reduced FMN molecule which is not consumed during the reaction. In the elimination reaction the most accepted mechanism suggests a direct role of reduced FMN that transfers the electron to phosphate and the substrate donates an electron for the regeneration of FMN. Furthermore, the monofunctional form of chorismate synthase is found in plants and bacteria whereas in bi-functional it occurs in fungi [3].

Characterization of this pathway in bacteria was achieved largely by studying mutants lacking the individual enzyme activities. Shikimate pathway is essential in bacteria since enzymatic mutations in this pathway completely inhibit the growth in culture unless aromatic supplements are provided [10]. Studies of Barea and Giles showed that shikimate pathway in fungi play an essential role in synthesis of aromatic amino acid [11]. The genomic studies confirmed that this pathway could be efficiently used for the development of broad spectrum antimicrobial compound against variety of infectious diseases [10]. Earlier reports have shown that inhibition of one of the enzyme of shikimate pathway could efficiently treat the opportunistic pathogens such as *Pneumocystis carinii*, *Mycobacterium tuberculosis*, *Cryptosporidium parvum* and *Toxoplasma gondii*, which may simultaneously infect AIDS and other immune compromised patients [12].

A promising drug target for bacterial pathogenic diseases could be developed by blocking any enzymes of this pathway. Designing inhibitors for this reaction would greatly facilitate researchers to block multiple pathways essential for the survival of micro organism. The Chorismate Synthase Database provides data incorporating all the parameters required for the inhibition of chorismate synthase in 42 pathogenic bacterial species which is a potential drug target for blocking the shikimate pathway. A list of 48 inhibitors reported in literature with their IC50 and Ki values are shown in Table 1.

**Table 1:** List of chorismate synthase inhibitors

**Sl no**	**Pathogen name**	**Inhibitors name**	**IC50 nM**	**Ki nM**
1	*Escherichia coli*	(6R)-6-Fluoro-EPSP	500 and 250000	3000
2	*Salmonella typhimurium*	2-(3-(-(S)-5-((S)-1-Amino-3-(3-chlorophenyl)-1-oxopropan-2-ylamino)-4-(3-hydroxy-4-methyl-2-nitrobenzamido)-5-oxopentylcarbamoyl)-phenoxy)acetic Acid	n/a	720000
		2-(3-(3-((R)-3-((S)-1-Amino-3-(3-chlorophenyl)-1-oxopropan-2-ylamino)-2-(3-hydroxy-4-methyl-2-nitrobenzamido)-3-oxopropylthio) propylcarbamoyl)phenoxy)acetic Acid	n/a	2500000 ± 120000
		N-((S)-6-Amino-1-((S)-1-amino-3-(3-chlorophenyl)-1-oxopropan-2-ylamino)-1-oxohexan-2-yl)-3-hydroxy-4-methyl-2-nitrobenzamide	n/a	5700000 ± 1000000
		N-((S)-1-((S)-1-Amino-3-(3-chlorophenyl)-1-oxopropan-2-ylamino)-1-oxopropan-2-yl)-3-hydroxy-4-methyl-2-nitrobenzamide	n/a	6800000 ±2000000
3	*Streptococcuspneumoniae*	(2E)-6,7-dihydroxy-2-[(2-hydroxy-4-pentoxyphenyl)methylidene]-1-benzofuran-3- one	220.0	n/a
		(2E)-2-[(4-hexoxy-2-hydroxyphenyl)methylidene]-6, 7-dihydroxy-1-benzofuran-3-one	320.0	n/a
		(2E)-2-[(4-butoxy-2-hydroxyphenyl)methylidene]-6, 7-dihydroxy-1-benzofuran-3-one	450.0	n/a
		(2E)-6,7-dihydroxy-2-[(2-hydroxy-4-propoxyphenyl)methylidene]-1-benzofuran-3- one	510.0	n/a
		(2E)-6,7-dihydroxy-2-[[2-hydroxy-4-(2-methylpropoxy)phenyl]methylidene]-1- benzofuran-3-one	580.0	n/a
		ethyl 4-[4-[(E)-(6, 7-dihydroxy-3-oxo-1-benzofuran-2-ylidene)methyl]-3-hydroxyphenoxy] butanoate	650.0	n/a
		(2E)-6,7-dihydroxy-2-[(2-hydroxyphenyl)methylidene]-1-benzofuran-3-one	800.0	n/a
		(2E)-6,7-dihydroxy-2-[(2-hydroxy-3-methoxyphenyl)methylidene]-1-benzofuran-3- one	800.0	n/a
		(2E)-6,7-dihydroxy-2-[[2-hydroxy-4-(4-hydroxybutoxy)phenyl]methylidene]-1- benzofuran-3-one	860.0	n/a
		(2E)-6,7-dihydroxy-2-[(2-hydroxy-4-propan-2-yloxyphenyl)methylidene]-1- benzofuran-3-one	1000.0	n/a
		(2E)-6,7-dihydroxy-2-[(2-hydroxy-4-phenylmethoxyphenyl)methylidene]-1- benzofuran-3-one	1000.0	n/a
		4-[4-[(E)-(6,7-dihydroxy-3-oxo-1-benzofuran-2-ylidene)methyl]-3-hydroxyphenoxy] butanenitrile	1100.0	n/a
		4-[4-[(E)-(6,7-dihydroxy-3-oxo-1-benzofuran-2-ylidene)methyl]-3-hydroxyphenoxy] butanoic acid	1100.0	n/a
		(2E)-2-[(4-butoxyphenyl)methylidene]-6,7-dihydroxy-1-benzofuran-3-one	1500.0	n/a
		(2E)-6,7-dihydroxy-2-[[2-hydroxy-4-(6-hydroxyhexoxy)phenyl]methylidene]-1- benzofuran-3-one	1600.0	n/a
		(2E)-6,7-dihydroxy-2-[(2-hydroxy-4-methoxyphenyl)methylidene]-1-benzofuran-3- one	1700.0	n/a
		2-[2-[(E)-(6,7-dihydroxy-3-oxo-1-benzofuran-2-ylidene)methyl]phenoxy]acetic acid	1800.0	n/a
		(2E)-6,7-dihydroxy-2-[(2-nitrophenyl)methylidene]-1-benzofuran-3-one	2000.0	n/a
		(2E)-6,7-dihydroxy-2-[[2-hydroxy-4-(3-hydroxypropoxy)phenyl]methylidene]-1- benzofuran-3-one	2500.0	n/a
		2-[4-[(E)-(6,7-dihydroxy-3-oxo-1-benzofuran-2-ylidene)methyl]-3-hydroxyphenoxy]acetic acid	2600.0	n/a
		(2E)-2-[[4-[ethyl(2-hydroxyethyl)amino]-2-methylphenyl]methylidene]-6, 7-dihydroxy-1-benzofuran-3-one	3400.0	n/a
		(2E)-2-benzylidene-6,7-dihydroxy-1-benzofuran-3-one	3500.0	n/a
		(2E)-6,7-dihydroxy-2-[(2-methylphenyl)methylidene]-1-benzofuran-3-one	4000.0	n/a
		(2E)-6,7-dihydroxy-2-[(4-nitrophenyl)methylidene]-1-benzofuran-3-one	1700.0	n/a
		(2E)-2-[[4-(diethylamino)-2-hydroxyphenyl]methylidene]-6,7-dihydroxy-1-benzofuran-3-one	5000.0	n/a
		(2E)-6,7-dihydroxy-2-[(4-imidazol-1-ylphenyl)methylidene]-1-benzofuran-3-one	5100.0	n/a
		(2E)-2-[(2-chlorophenyl)methylidene]-6,7-dihydroxy-1-benzofuran-3-one	5200.0	n/a
		(2E)-6,7-dihydroxy-2-[(4-pyrrolidin-1-ylphenyl)methylidene]-1-benzofuran-3-one	5500.0	n/a
		(2E)-2-[(4-ethoxyphenyl)methylidene]-6,7-dihydroxy-1-benzofuran-3-one	5800.0	n/a
		(2E)-6,7-dihydroxy-2-[[4-(trifluoromethyl)phenyl]methylidene]-1-benzofuran-3- one	7000.0	n/a
		(2E)-2-[[4-(diethylamino)phenyl]methylidene]-6,7-dihydroxy-1-benzofuran-3-one	8500.0	n/a
		ethyl 2-[4-[(E)-(6, 7-dihydroxy-3-oxo-1-benzofuran-2-ylidene)methyl]-3-hydroxyphenoxy] acetate	10300.0	n/a
		2-[(E)-(6,7-dihydroxy-3-oxo-1-benzofuran-2-ylidene)methyl]benzoic acid	10700.0	n/a
		(2E)-6,7-dihydroxy-2-[(4-methoxyphenyl)methylidene]-1-benzofuran-3-one	14300.0	n/a
		(2E)-6,7-dihydroxy-2-[(4-hydroxyphenyl)methylidene]-1-benzofuran-3-one	15000.0	n/a
		(2E)-6,7-dihydroxy-2-[[2-(trifluoromethyl)phenyl]methylidene]-1-benzofuran-3- one	17300.0	n/a
		4-[(E)-(6,7-dihydroxy-3-oxo-1-benzofuran-2-ylidene)methyl]benzoic acid	24000.0	n/a
		(2E)-2-[[4-(dimethylamino)phenyl]methylidene]-6, 7-dihydroxy-1-benzofuran-3-one	25900.0	n/a
		(2E)-6,7-dihydroxy-2-[(4-morpholin-4-ylphenyl)methylidene]-1-benzofuran-3-one	>50000.0	n/a
		(2E)-2-[(4-fluorophenyl)methylidene]-6,7-dihydroxy-1-benzofuran-3-one	>60000.0	n/a
		(2E)-6,7-dihydroxy-2-[(2-methoxyphenyl)methylidene]-1-benzofuran-3-one	>60000.0	n/a
		[(2S,3R,4R)-5-(7,8-dimethyl-2,4-dioxobenzo[g]pteridin-10-yl)-2,3, 4-trihydroxypentyl] dihydrogen phosphate	n/a	n/a

## Construction and content

### Data sources and curation

The starting point for data curation in Chorismate Synthase Database is a manual curation of all publicly available sequence, structure and functional information for pathogens from UniProtKB [13,14]. Other database identifiers (e.g. NCBI taxonomy codes, Gene Ontology classifications, InterPro and Pfam accessions, super family, SCOP, prosite, KEGG, Pubchem Substance, etc.,) were also imported apart from the literature references, annotations of sequence and structure features. CSDB taxonomy is derived from the NCBI taxonomy database.

**Figure 1:**
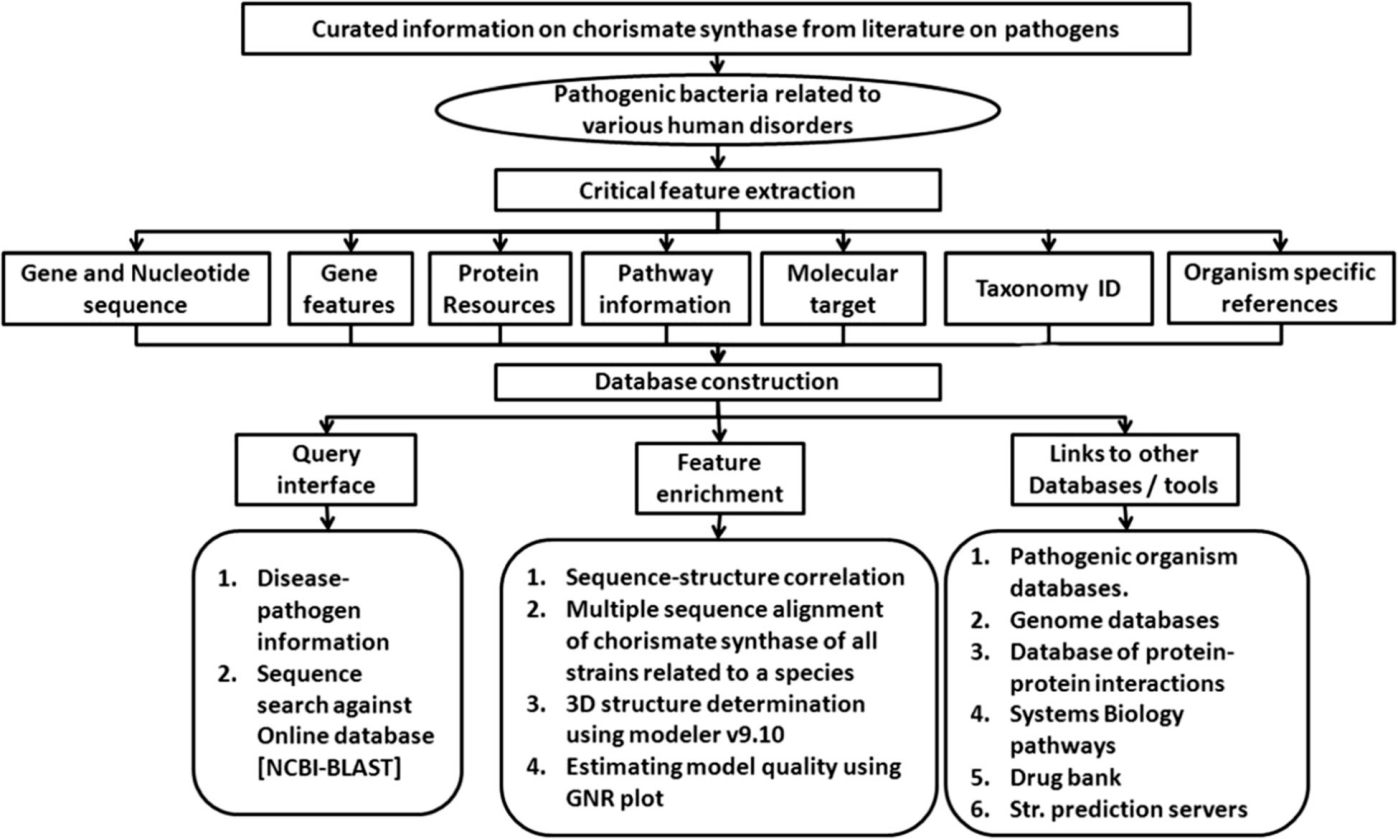
Structure of Chorismate synthase database.

The data in CSDB is organized into 7 fields (Figure 1) such as protein resources, gene annotations, features, gene and nucleotide sequence, pathways, molecular target, taxonomical ID and literature references. The classification of pathogenic bacteria used in CSDB is similar to that of the already available pathogenic bacteria listed in “Classification of Pathogenic Bacteria” available at the weblink (http://www.buzzle.com/articles/pathogenic-bacteria-list.html). Links are provided to access further information on the Pathogenic Bacteria, if present in external databases like *Swiss-Prot, NCBI* Taxonomy Browser, EMBL-EBI, Sanger institute, chemical database, PDB and Pubmed reference etc.,

An extensive literature survey was carried out using PUBMED and MEDLINE to extract information about human diseases caused by various bacterial pathogens. Critical features related to chorismate synthase for each bacterial strain such as gene sequence, gene id, protein sequence in fasta format, domain and motif information were retrieved from domain and motif databases. The structure related information were retrieved from PDB, CATH, and SCOP, kinetic data from literature, pathway information from KEGG, and its Gene Ontology information were retrieved from GO database. A database was constructed using these information by integrating them appropriately in a flat file format.

The features of this database can be categorized in to three broad areas:

1. Query interface: The query interface is a collection of all the pathogenic bacteria with their strain information available in literature and relates to the disease it causes to humans.

2. Feature enrichment: Feature enrichment category is sequence annotation from well curated databases, multiple sequence alignment in chorismate synthase of all strains and 3D structure determination using Modeller v.9.10 and its validation using GNR plot.

3. External references/links: This category includes pathogenic organism database, Genome databases, Database of protein-protein interactions, Systems Biology pathways, Drug bank and Structure prediction servers.

**Figure 2:**
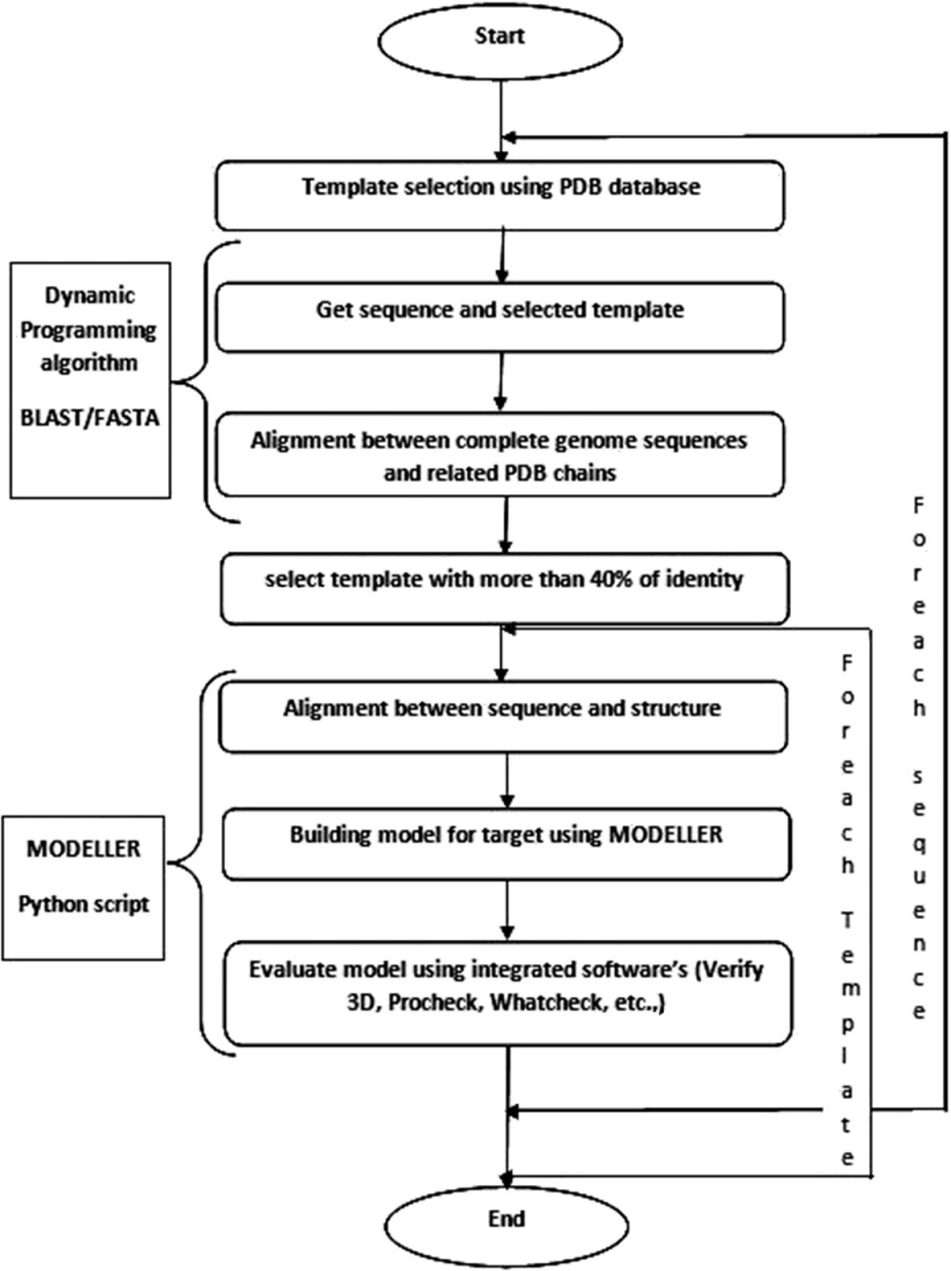
Schematic workflow for homology modeling.

The molecular modeling in this work was performed by the MODELLER version 9.10. The MODELLER program was completely automated to calculate comparative models for a large number of protein sequences, by using many different template structures and sequence-structure alignments [15-17]. Sequence-structure matches are established by aligning SALIGN [18,19]. Sequence profile of the target sequence against each of the template sequences extracted from PDB [14] (Figure 2).

### Database architecture

**Figure 3:**
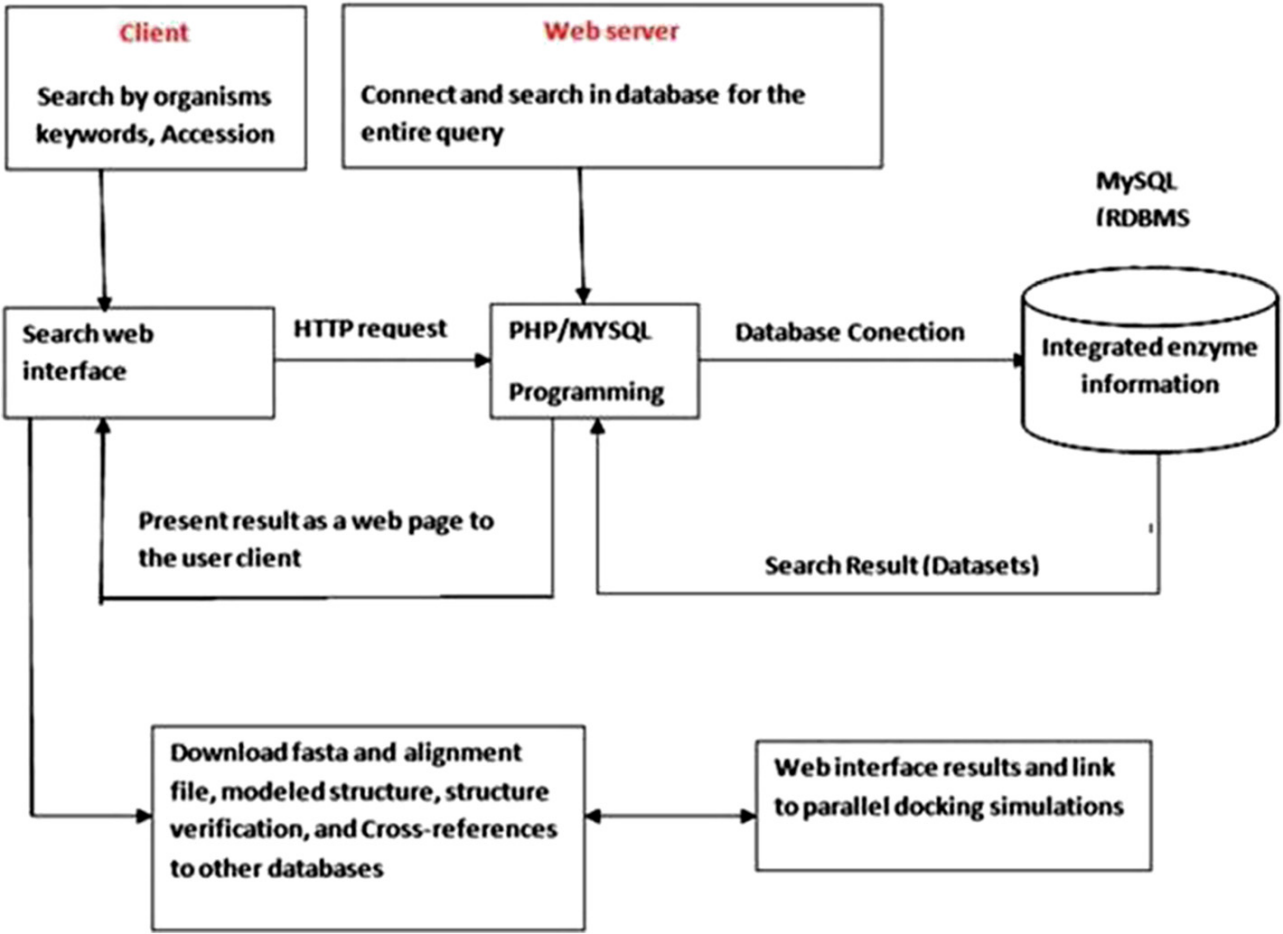
Schematic draw showing the Interaction of web client interface.

CSDB is built on Apache HTTP Server 2.2.11 with MySQL Server 5.1.36 as the back-end and PHP 5.3.0, HTML and JavaScript, CSS as the front-end. Apache, MySQL and PHP technology were preferred as they are open-source software’s and platform independent. Besides these advantages, MySQL is the most popular open source SQL (Structured Query Language) database over the internet. MySQL (Figure 3) is a relational database management system that works much faster which also supports multi-user and multi-threading. It can work both on Windows and Linux. It comes with Triggers, Cursors and stored procedures to improve the productivity of developers.

## Utility and discussion

### Data access

Data stored in CSDB can be accessed in the following ways: (i) Search options in CSDB: CSDB can be queried to obtain pathogen information. In order to facilitate this, simple search options or manual browse option have been provided in the ‘Search’ section.

**Figure 4:**
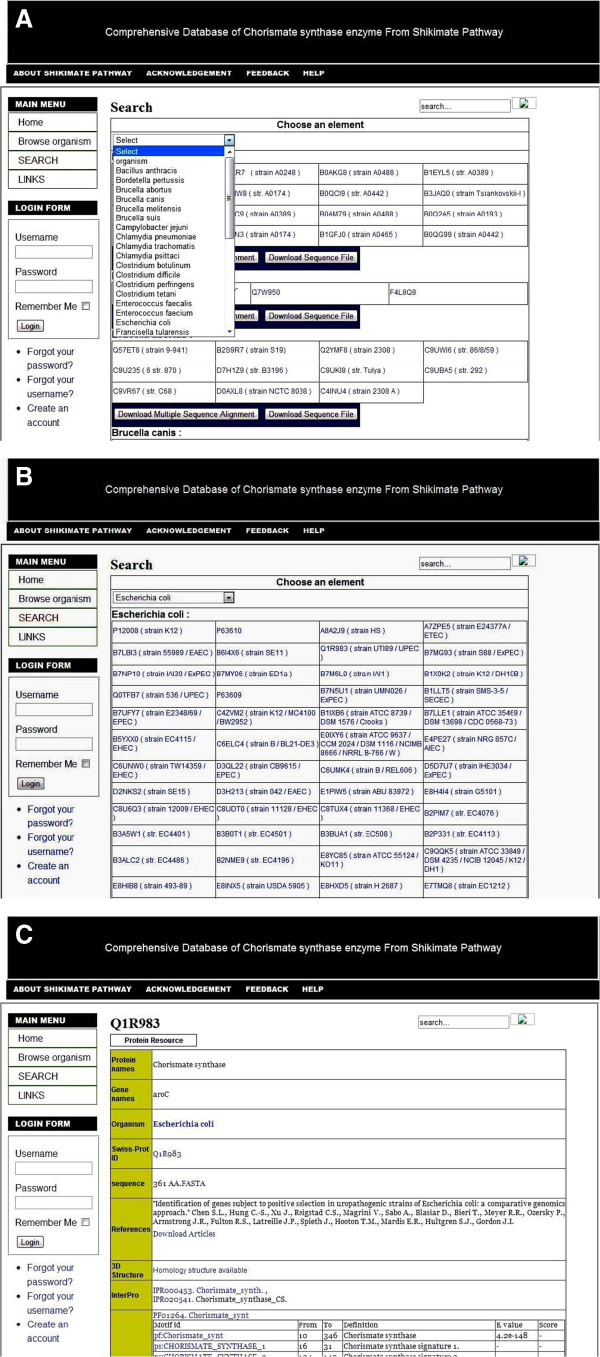
**Chorismate synthase database search section.** (**A**) Organism based selection. (**B**) The list of proteins found in a selected organism. (**C**) The list of selected protein with their major features.

Select pathogenic bacteria: the user can select pathogenic bacteria to obtain related information on bacteria. (Figure 4) illustrates the result of organism-based search).

### External links

External database links are provided in the web portal by using hyperlinks to other useful bioinformatics resources such as genome database, protein-protein interactions databases, system biology pathways, pathogenic organism databases, microarray databases, structure prediction server and GENE CARDS.

#### Feedback

Users can submit their suggestions/comments/queries using this feature.

#### Help

A detailed description on the use of the various features incorporated in CSDB is provided in this section for the benefit of users.

### Future work

The resource will be updated constantly with further enhanced features. We also intend to add some bioinformatics tools on structural and sequence analysis in future versions. We would also like to extend this database for other pathogens.

## **Conclusions**

The CSDB provides manually curated information on analysis of chorismate synthase in 42 pathogenic bacterial species. This database provides information useful for designing a drug in both ligand as well as structure based methods. For structure based drug design, information on the protein’s motif and Interpro’s/PFAM domain categorization are been added and 48 inhibitors with IC50/Ki values are made available for designing inhibitors using Ligand based drug design strategies [Table 1]. In addition to this, this database also contains information about the protein’s superfamily, SCOP IDs, GO IDs, active site residues pathway information using KEGG, taxonomy, and structural models using modeler 9.10. This facilitates their usage in drug design for researchers. This database is freely available at the website http://www.csdb.in.

### Availability and requirements

CSDB is freely available at http://www.csdb.in.

### Download

CSDB database contents can be downloaded easily from the ‘Download Database’ section. Users can obtain the entire collection of ID’s at SQL format with a single mouse click.

## Competing interests

The authors declare that they have no competing interests.

## Authors’ contributions

PP participated in the literature search, data curation, constructing the database and manuscript preparation. WH and RR participated in the final analysis, interpretation and preparation of the manuscript. All authors read and approved the final manuscript.

## Pre-publication history

The pre-publication history for this paper can be accessed here:

http://www.biomedcentral.com/2050-6511/14/29/prepub
